# Clearance of mannitol for assessment of glomerular filtration rate in chronic kidney disease: A validation against iohexol clearance

**DOI:** 10.1111/cpf.70059

**Published:** 2026-04-03

**Authors:** Katalin Kiss, Aso Saeed, Sven‐Erik Ricksten, Gudrun Bragadottir

**Affiliations:** ^1^ Department of Anesthesiology and Intensive Care Medicine, Institute of Clinical Sciences, Sahlgrenska Academy University of Gothenburg Gothenburg Sweden; ^2^ Department of Molecular and Clinical Medicine/Nephrology, Institute of Medicine, Sahlgrenska Academy University of Gothenburg Gothenburg Sweden

**Keywords:** chronic kidney disease, glomerular filtration rate, iohexol, mannitol, plasma clearance

## Abstract

This study evaluates mannitol as a clearance marker for measuring glomerular filtration rate (GFR) in outpatients with chronic kidney disease (CKD). Multi‐sample iohexol clearance served as the reference method. Twenty patients with CKD, stages 3–4, received bolus injections of both clearance markers simultaneously. Blood samples were collected 3, 5 and 22 h after the bolus injections. Marker concentrations were analyzed using liquid chromatography‐tandem mass spectrometry (LC‐MS/MS). A calculation template, based on the Bröchner–Mortensen multi‐sample model, was applied to determine GFR for both markers. In addition, plasma clearance of mannitol was assessed using the one‐sample Jacobsson equation, applied to samples taken 5 and 22 h after the bolus injection of mannitol. Agreement between the clearance methods was assessed by the Bland‐Altman method, and the accuracy of mannitol clearance versus iohexol clearance was assessed by calculating P30 and P10. For the multi‐sample clearance, the between‐methods bias was 0.95 ± 1.4 mL/min/1.73 m², the error was 10%, and the accuracy was 100% for both P30 and P10. The corresponding values for bias, error, P30 and P10 for the one‐sample plasma clearance of mannitol versus the reference method were: −2.3 ± 3.6 and 2.9 ± 3.0 mL/min/1.73 m², 24% and 21%, 95% and 100%, 80% and 60%, respectively for samples obtained 5 and 22 h after the bolus. Mannitol exhibited a reliable performance as a clearance marker in patients with CKD, stages 3–4, and may serve as an alternative when standard markers, iohexol or isotopes, are contraindicated. However, further large‐scale studies are required to confirm these findings.

## 1 INTRODUCTION

Assessment of glomerular filtration rate (GFR) is a fundamental measure of kidney function. It is essential for detecting kidney diseases, classifying disease stages, and monitoring progression of kidney failure in patients with chronic kidney diseases (CKD) (Bellomo et al., 2012; Cockcroft & Gault, 1976; Jones et al., 1998; Levey, 1990). Estimated GFR (eGFR), derived from serum creatinine and/or cystatin C‐based equations, constitutes the cornerstone of CKD diagnosis and management (Inker et al., 2014; Inker et al., 2021; Tangri et al., 2016). Beyond its role in assessing kidney function, eGFR, particularly cystatin C‐based eGFR, has emerged as an important predictor of cardiovascular morbidity and mortality (Jernberg et al., 2004; Shlipak et al., 2005). However, eGFR is a population‐derived estimate that cannot fully account for individual biological variability. Serum creatinine is influenced by non‐GFR determinants such as muscle mass and diet, while cystatin C may be affected by factors including thyroid disease and corticosteroid use, resulting in different sources of bias (Manetti et al., 2005; Risch et al., 2001). Although equations combining creatinine and cystatin C equations improve accuracy at the population level, clinically relevant imprecision persists, particularly at GFR decision thresholds and extremes of age or kidney function (Grubb et al., 2014; Inker et al., 2021; Pottel et al., 2023; Stevens et al., 2006). These potential limitations highlight the continued importance of measured GFR using exogenous substances as filtration markers, which provides a direct and unbiased assessment and remains essential for high‐precision clinical decisions, research, and validation of estimating equations.

Measurement of GFR by using exogenous substances as filtration markers, such as inulin, iohexol, ^123^I‐iothalamate, diethylene triamine pentaacetic acid (Tc‐99 m DTPA), and chromium‐ethylenediaminetetraacetic acid (^51^Cr‐EDTA), is considered as the standard approach for assessment of kidney function (Brandstrom et al., 1998; Delanaye, Ebert, et al., 2016; Delanaye, Melsom, et al., 2016; Fleming et al., 1991; Krutzén et al., 1984; Malamos et al., 1967).

Plasma clearance of inulin is regarded as the gold standard for GFR measurement. However, it is technically demanding, time‐consuming, and not practical for routine clinical use (Blaufox et al., 1996). In addition, inulin has become increasingly difficult to obtain and is today replaced by single bolus plasma clearance methods using either the isotope ^51^Cr‐EDTA or the non‐radioactive radiocontrast agent, iohexol (Brandstrom et al., 1998; Brown & O′Reilly, 1991; Delanaye, Ebert, et al., 2016; Delanaye, Melsom, et al., 2016; Soveri et al., 2014).

Mannitol meets the criteria for an optimal clearance marker (Elkinton, 1947; Schwartz et al., 1950; Smith et al., 1940; Smith et al., 1945). Our research group has previously compared the performance of plasma clearance of mannitol to the standard ^51^Cr‐EDTA clearance for measuring GFR showing that there was a high agreement between the two methods (Kiss et al., 2018). In that study, multi‐sample plasma clearance of mannitol was compared with single‐sample clearance of ^51^Cr‐EDTA according to the Jacobsson method (Jacobsson, 1983). The radioactive filtration marker, ^51^Cr‐EDTA, is, however, no longer available in our country. Furthermore, the GFR of the outpatients included in the study ranged from normal to moderately reduced, and mannitol concentrations in serum were determined using an enzymatic method that has been shown to be impractical for routine clinical use (Graefe et al., 2003).

The aim of the present study was to evaluate the agreement between multi‐sample and single‐sample plasma clearances of mannitol and the reference method, multi‐sample plasma clearance of iohexol, for assessment of GFR in patients with moderate to advanced CKD (CKD stages 3–4). Plasma concentrations of iohexol and mannitol were determined using liquid chromatography‐tandem mass spectrometry (LC‐MS/MS) (Annesley & Clayton, 2009; Deferm et al., 2021). We hypothesized that mannitol clearance would show good agreement with the reference method, multi‐sample clearance of iohexol, for the assessment of kidney function.

## 2 METHODS

The study was conducted in accordance with the principles of the Declaration of Helsinki and the Vancouver recommendations. The study protocol was approved by the Gothenburg Regional Ethical Committee (https://etikprovningsmyndigheten.se) and the Swedish Ethical Review Authority (https://etikprovningsmyndigheten.se), (protocol: 745–09, 640‐16) (protocol: 745–09, 640‐16) and was registered in ClinicalTrials.gov (NCT06238310). Before enrolment in the study, the patients were informed about the objectives and procedures of the study. Informed consent was obtained from each patient.

### 2.1 Study participants

Patients were recruited from the Nephrology Outpatient Clinic at Sahlgrenska University Hospital, Gothenburg, Sweden, between November 2023 and February 2024. Patients with a planned follow‐up within 3 months were invited to participate, and a total of 20 patients (10 women and 10 men) were enrolled. Inclusion criteria included age >18 years and an estimated GFR of 15–59 mL/min/1.73 m², as determined by the Chronic Kidney Disease Epidemiology Collaboration (CKD‐EPI) equation based on creatinine, for at least 3 months (i.e., CKD stages 3 and 4) (Charles et al., 2024). Exclusion criteria included a history of organ transplantation or ongoing immunosuppressive treatment for inflammatory systemic disease.

### 2.2 Experimental procedure

Multi‐sample plasma clearance measurements using the bolus injection technique were performed with mannitol and iohexol. Two blank samples were drawn at the start of the study, to measure plasma concentrations of mannitol and iohexol before bolus injections (*T*_0_). Thereafter, a bolus dose of mannitol, 0.25 g/kg (Mannitol Baxter Viaflo ®) was administered using a volumetric infusion pump, followed by a bolus dose of iohexol (Omnipaque 300 mg/mL, GE Healthcare), 5 mL for patients weighing over 50 kg and 4 mL for those weighing less than 50 kg. Three additional blood samples were collected, 3, 5 and 22 h after the initial doses of mannitol and iohexol to measure the declining plasma concentrations of the markers. Lithiumheparin tubes were used for collecting blood samples for the measurements of mannitol concentrations, while EDTAtubes were used for samples for the measurements of iohexol concentrations.

Blood samples were gently mixed by inverting them 10 times, and then they were kept at room temperature for 30 min before centrifugation at 2000 *g* for 10 min at +4°C. The supernatant was transferred into Eppendorf tubes, immediately frozen, and stored at −80°C until analysis. The exact time of sample collection was recorded for both markers.

Mannitol plasma concentrations were analyzed at the Ardena Laboratory, Assen, The Netherlands, while iohexol concentrations were analyzed at the Laboratory for Clinical Chemistry, Sahlgrenska University Hospital, Gothenburg, Sweden. The analyses of mannitol (see Supplemental File 1) and iohexol were performed using LC‐MS/MS (Annesley & Clayton, 2009).

### 2.3 Calculation of plasma clearance for mannitol and iohexol

Plasma concentrations for both markers were expressed in mg/L. A calculation template, owned and used by the Laboratory of Clinical Chemistry at the Sahlgrenska University Hospital, was applied to determine GFR for both markers (GFR_mannitol_ and GFR_iohexol_). The template is based on the multi‐sample calculation model by Bröchner‐Mortensen (Bröchner‐Mortensen, 1972) (see Supplemental File 2). Plasma clearance for mannitol was also assessed by the one‐sample Jacobsson technique (Jacobsson, 1983). The samples were taken 5 and 22 h after the bolus injection of mannitol. A calculation template, owned and used by the Laboratory of Clinical Chemistry at the Sahlgrenska University Hospital based on the one‐sample calculation model by Jacobsson was applied to determine GFR for mannitol (Jacobsson, 1983).

### 2.4 Statistical analysis

Values are presented as means ± standard deviation (SD) unless stated otherwise. A regression analysis was performed to assess correlations between individual data on iohexol clearance ad modum Bröchner–Mortensen (reference method) and mannitol clearance assessed by the multi‐ and the two single‐sample approaches. The agreement between GFR_mannitol_ and GFR_iohexol_ was assessed according to Bland and Altman (Bland & Altman, 1986). The mean difference between two methods (bias) and the standard deviation of the differences were calculated, as well as the error (double standard deviation divided by the mean of the measurements from the two methods) and the limits of agreement (mean difference ± two standard deviations). According to Critchley and Critchley, an acceptable between‐method error was defined as 30% or less (Critchley & Critchley, 1999).

Furthermore, the accuracy of GFR_mannitol_ was assessed as the proportion of estimates within 30% (P30) and 10% (P10) of GFR_iohexol_ in both patient groups. It is recommended that the P30 and P10 values should be at least 80% and 50%, respectively, for a new method to be acceptable (Soveri et al., 2014).

SPSS Statistics 29 (IBM) was used for all the statistical analyses. Statistical significance was set at the level of *p* < 0.05.

## 3 RESULTS

Twenty patients (10 women and 10 men) with CKD stages 3–4 were included in the study after providing informed consent. The characteristics of the study participants at inclusion are presented in Table 1. The mean age was 69 ± 6 years and mean body surface area (BSA) was 2.0 ± 0.2 m^2^. Fifty‐five percent of the patients had CKD stage 3%, and 45% had CKD stage 4. In their previous medical history, the following diagnoses were the most common: hypertension (100%), diabetes (20%) and obesitas (35%). The mean plasma creatinine for the group was 163 ± 47 µmol/L.

**TABLE 1:** Characteristics of the study participants at inclusion (*n* = 20).

Female *n* (%)	10 (50)
Male *n* (%)	10 (50)
Age	69 ± 6
Body height (cm)	171 ± 11
Body weight (kg)	82 ± 15
Body surface area (m^2^)	2.0 ± 0.22
Body mass index (kg/m^2^)	27.96 ± 5.01
Plasma creatinine (µmol L^−1^)	163 ± 47
Plasma cystatin C (mg/L)	2.16 ± 0.57
Plasma urea (mmol/L)	13 ± 7
Plasma albumin (g/L)	36.8 ± 4.4
Chronic kidney disease stage 3 *n*, (%)	11 (55)
Chronic kidney disease stage 4 *n*, (%)	9 (45)
Hypertension *n*, (%)	20 (100)
Diabetes *n*, (%)	4 (20)
Obesitas *n*, (%)	7 (35)

*Note*: Data are means ± SD unless stated otherwise.

### 3.1 Correlation between GFR_mannitol_ and GFR_iohexol_

**FIGURE 1:**
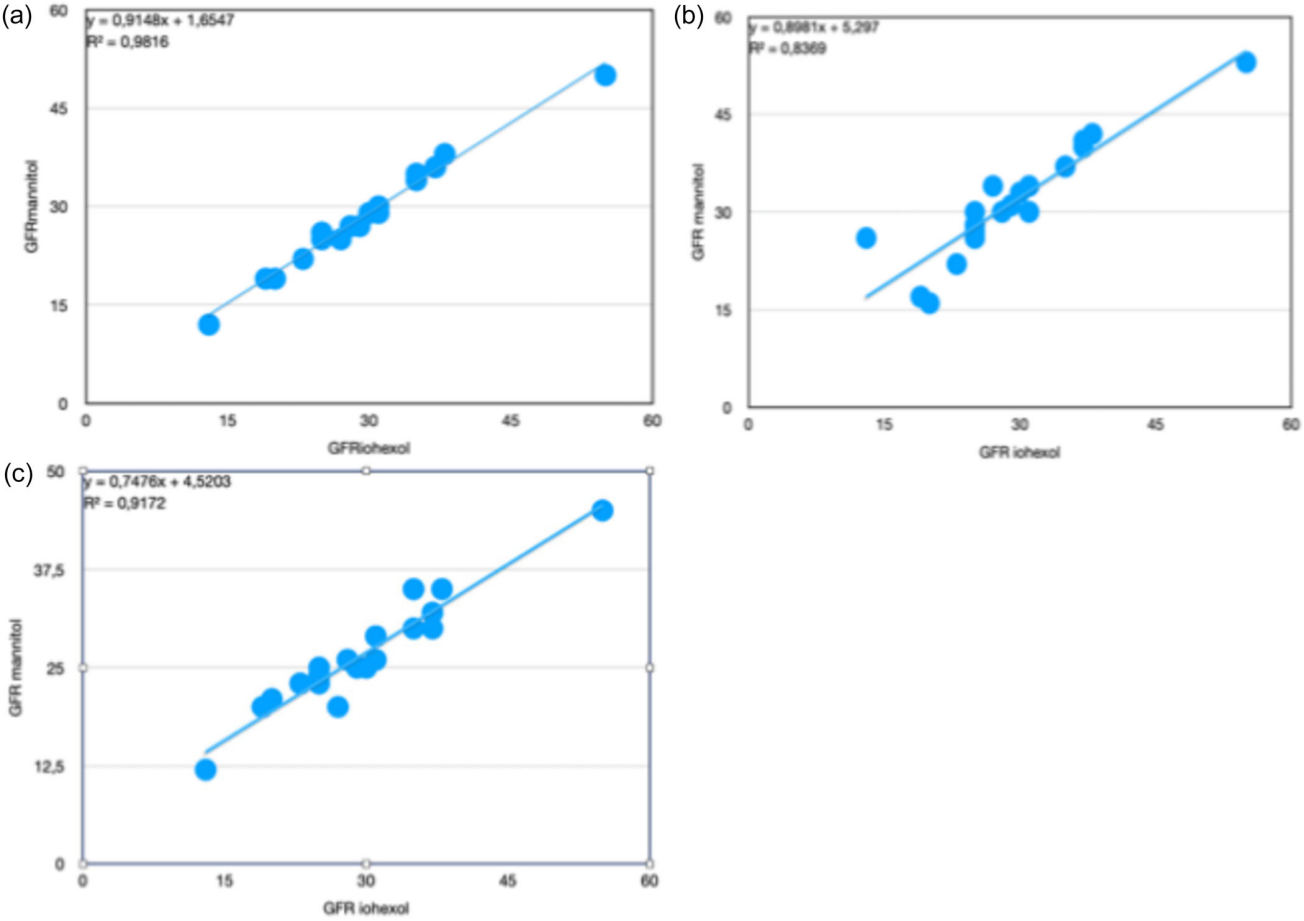
(a)–(c) Shows the correlation between GFR_iohexol_ and GFR_mannitol_ assessed by (a) the Brøchner–Mortensen multi‐sample approach, (b) the Jacobssons method for samples taken at 5 h and (c) the Jacobssons method for samples taken at 22 h.

There was a strong correlation between GFR_mannitol_ and GFR_iohexol_, calculated according to the Brøchner‐Mortensen method (*r* = 0.991) (Figure 1a). Similarly, strong correlations were observed between GFR_mannitol_ and GFR_iohexol_ calculated using the Jacobssons method for plasma mannitol samples obtained at 5 h (*r* = 0.915) and 22 h (*r* = 0.958) (Figure 1b and 1c, respectively).

### 3.2 Bias, error and accuracy of GFR_mannitol_ versus GFR_iohexol_ (multi‐sample Bröchner–Mortensen method)

**FIGURE 2:**
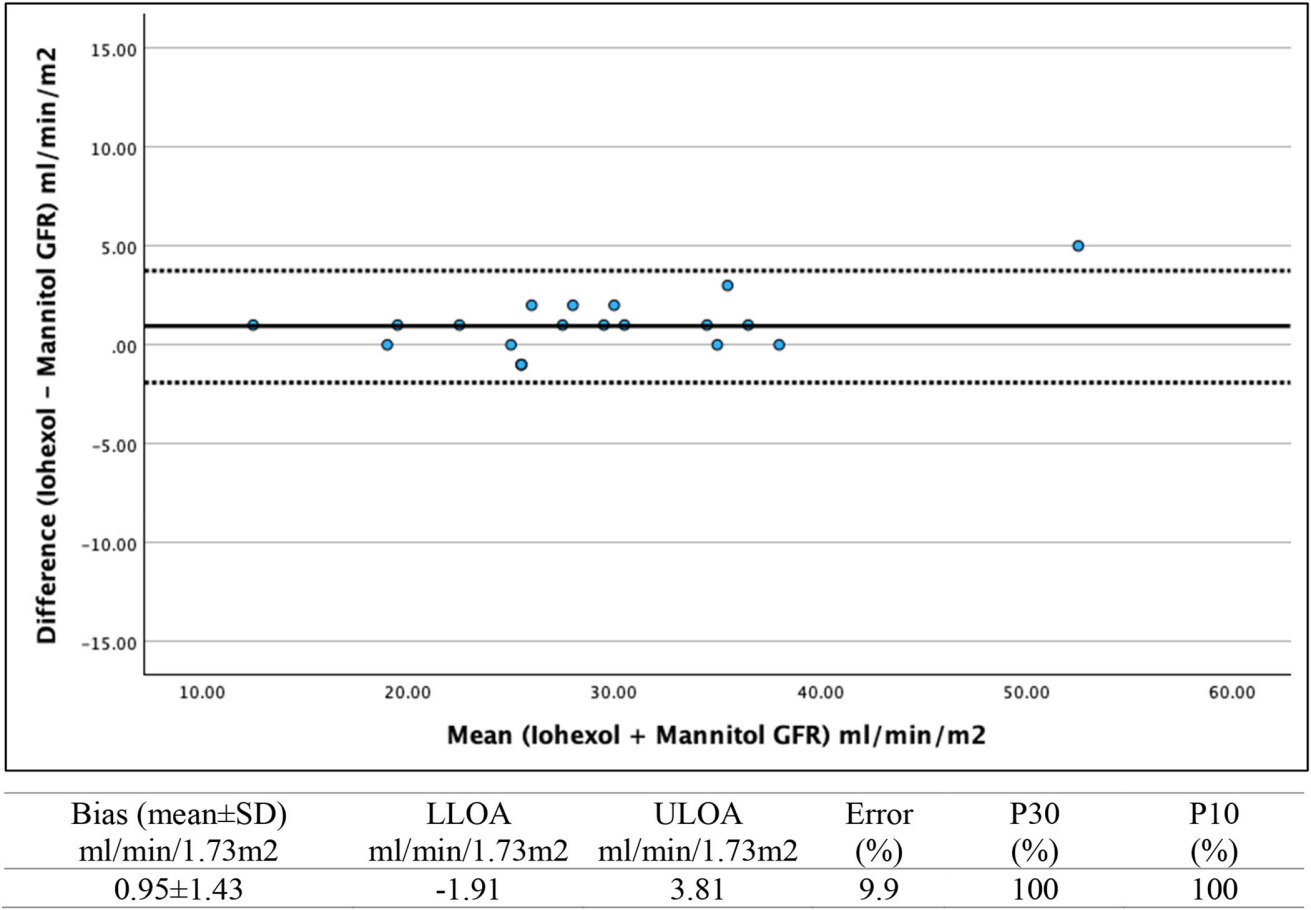
Bland–Altman plot for the agreement between GFR_mannitol_ and GFR_iohexol_ as the reference method using the Bröchner–Mortensen multi‐sample method for both markers (*n* = 20). The solid line represents the mean difference (±1.96 SD) (bias) between methods, and the dashed lines represent the upper (ULOA) and lower (LLOA) limits of agreement (±1.96 SD). P30 and P10 represent the proportion of GFR_mannitol_ estimates within 30% (P30) and 10% (P10) of GFR_iohexol_.

The mean bias value and Bland–Altman 95% limits of agreement between GFR_mannitol_ and GFR_iohexol_, are described in Figure 2. The mean bias was 0.95 ± 1.43 mL/min/1.73 m² and the error of GFR_mannitol_ was 9.9%. The accuracy was 100% for both P30 and P10.

### 3.3 Bias, error and accuracy of GFR_mannitol_ versus GFR_iohexol_ (one‐sample Jacobsson method)

**FIGURE 3:**
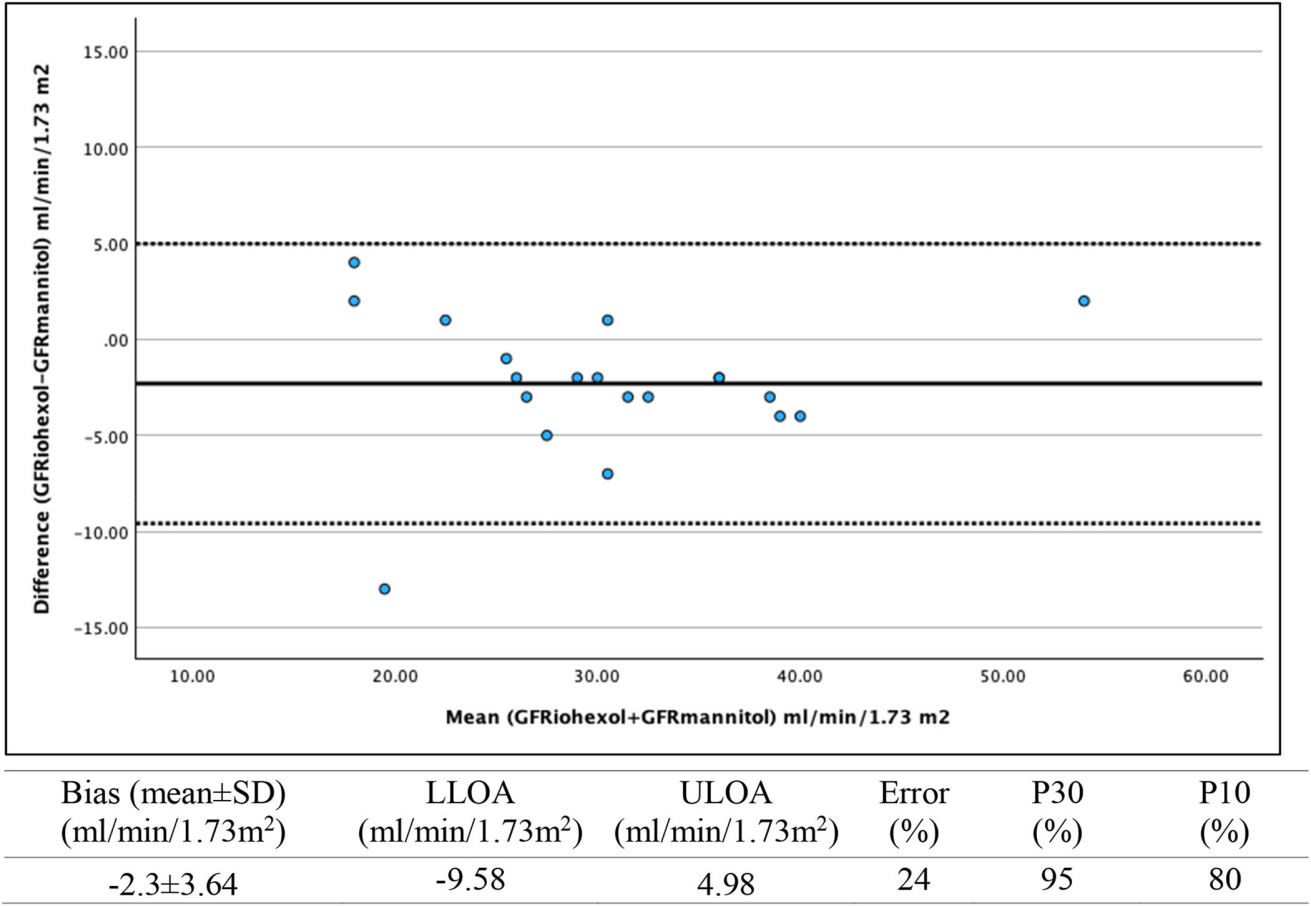
Bland–Altman plot for the agreement between GFR_mannitol_ calculated from the one‐sample Jacobsson method taken at 5 h and GFR_iohexol_ as the multi‐sample reference method (*n* = 20). The solid line represents the mean difference (±1.96 SD) (bias) between methods, and the dashed lines represent the upper (ULOA) and lower (LLOA) limits of agreement (±1.96 SD). P30 and P10 represent the proportion of GFR_mannitol_ estimates within 30% (P30) and 10% (P10) of GFR_iohexol_.

**FIGURE 4:**
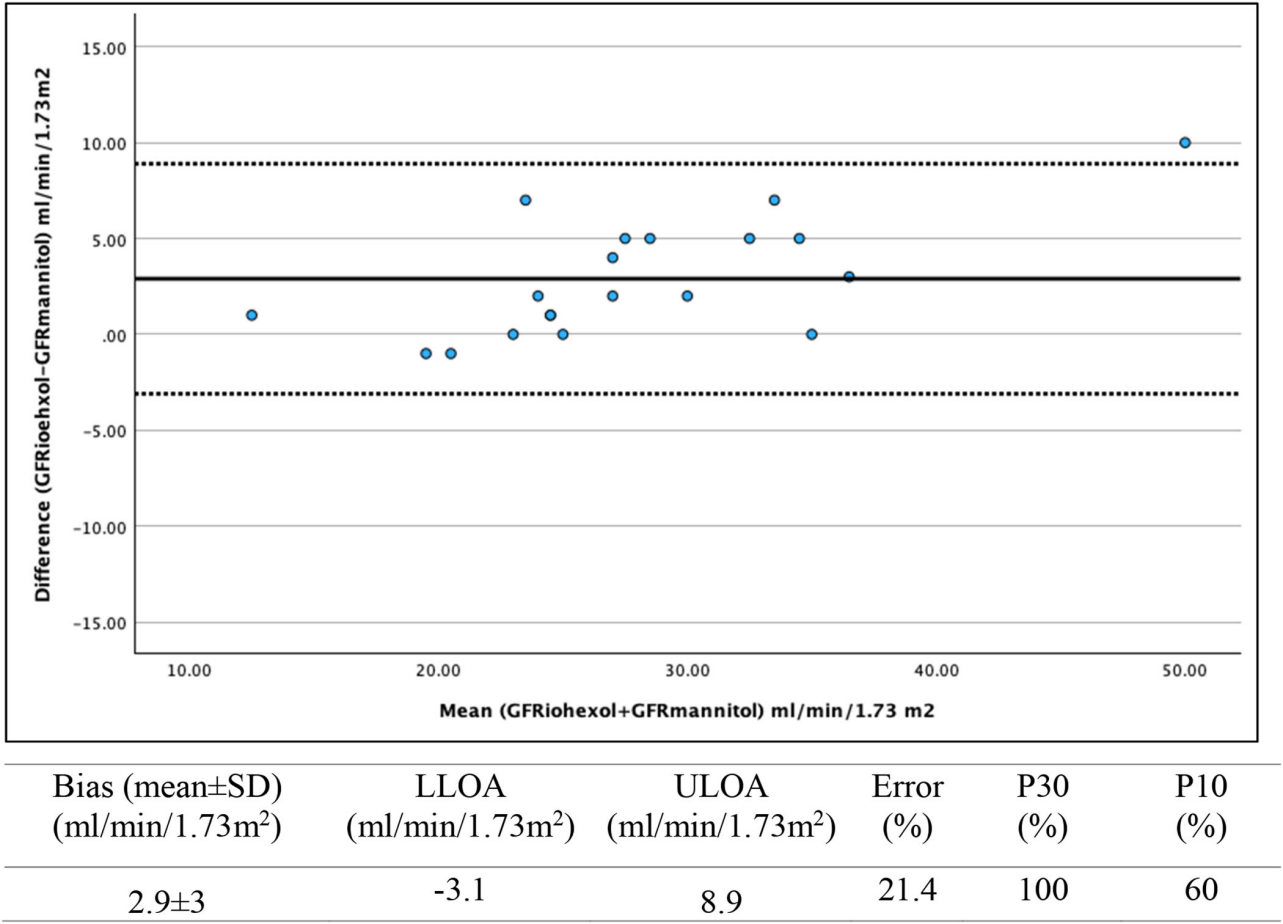
Bland–Altman plot for the agreement between GFR_mannitol_ calculated from the one‐sample Jacobsson method taken at 22 h and GFR_iohexol_ as the multi‐sample reference method (*n* = 20). The solid line represents the mean difference (±1.96 SD) (bias) between methods, and the dashed lines represent the upper (ULOA) and lower (LLOA) limits of agreement (±1.96 SD). P30 and P10 represent the proportion of GFR_mannitol_ estimates within 30% (P30) and 10% (P10) of GFR_iohexol_.

The mean bias value and Bland–Altman 95% limits of agreement between GFR_mannitol_ and GFR_iohexol_, are described in Figures 3 and 4. For plasma mannitol samples obtained at 5 h, the mean bias was −2.3 ± 3.6 and the error was 24%. The accuracy was 95% for P30 and 80% for P10 (Figure 3). For plasma mannitol samples obtained at 22 h, the mean bias was 2.9 ± 3.0 mL/min/1.73 m² and the error was 21%. The accuracy was 100% for P30 and 60% for P10 (Figure 4).

## 4 DISCUSSION

In the present study, we evaluated the use of mannitol clearance for GFR assessment in outpatients with stable CKD stages 3–4, using multi‐sample iohexol clearance as a reference method. The main findings of the study were that there was good agreement between multi‐sample iohexol clearance and the multi‐sample‐, as well as the one‐sample mannitol clearances obtained 5 and 22 h after bolus injection of iohexol and mannitol.

Critchley and Critchley have suggested an error ≤30% for an acceptance of a new method compared with a standard method (Critchley & Critchley, 1999). Furthermore, Soveri et al, have suggested that a new GFR measurement method (index method) should be considered to have an acceptable agreement with a reference method provided that at least 80% of the index measurements are within ±30% of the reference method (P30) and that at least 50% are within ±10% of the reference method (P10) (Soveri et al., 2014). In the present group of stable CKD patients, multi‐sample GFR_mannitol_ demonstrated excellent agreement with the reference method, with a very low bias (0.95 mL/min 1.73 m^2^) and error (9.9%), and perfect accuracy for both P30 and P10. Interestingly, good agreement with low biases and errors and high accuracy was observed when GFR_mannitol_ was calculated using the Jacobssons one‐sample method, particularly for samples taken 5 h after the bolus dose of mannitol. The observed bias was small, and the accuracy remained high with P30 of 95% and P10 of 80%. These findings suggest that one‐sample mannitol clearance, especially when measured at 5 h, might provide a reliable and practical estimate of GFR in patients with moderate to advanced CKD. The use of a single‐sample approach would simplify the procedure substantially, reducing costs, the number of blood draws and analytical workload, while maintaining clinically acceptable accuracy.

The gold standard method for assessment of GFR is plasma clearance of inulin (Blaufox et al., 1996). However, inulin clearance is not practical for routine clinical use and is today replaced by single bolus plasma clearance methods using either the isotope ^51^Cr‐EDTA or the non‐radioactive radiocontrast agent, iohexol (Blaufox et al., 1996; Brown & O′Reilly, 1991; Delanaye, Ebert, et al., 2016; Delanaye, Melsom, et al., 2016). The use of radioactive filtration markers like ^51^Cr‐EDTA requires access to a nuclear medicine department and should not be utilized in early pregnancy. Iohexol, a non‐radioactive contrast agent may require precautionary measures in diabetic patients with impaired kidney function who are being treated with metformin, due to the risk of lactic acidosis (Thomsen & Morcos, 1999). Furthermore, iohexol should be used with caution in patients with a known hypersensitivity to radiocontrast agents. Another disadvantage of iohexol clearance is that it cannot be used for 5 days after radiological examinations and interventions involving contrast agents.

Mannitol clearance has been shown to be identical to inulin clearance in studies performed in the 1940s and 1950s (Schwartz et al., 1950; Smith et al., 1940). However, difficulties in measuring serum mannitol, limited its use in clinical practice. Our research group has previously compared the performance of plasma clearance of mannitol, using the Bröchner–Mortensen approach, to the standard ^51^Cr‐EDTA clearance for measuring GFR, in outpatients with normal to moderately impaired kidney function (Kiss et al., 2018). The study showed that there was a high agreement between the methods, expressed as a low bias, acceptable error and high accuracy, particularly when using a three‐ or four‐point plasma disappearance curve for mannitol, obtained at 1 h intervals, and when mannitol measurements were started 60 min after the bolus of mannitol (Kiss et al., 2018). In that study, mannitol concentrations in plasma were measured by an enzymatic method that has been proved impractical for routine clinical use (Graefe et al., 2003).

Today, mannitol concentrations in plasma can be measured with LC‐MS/MS, one of the most accurate analytical methods available for plasma concentration measurements (Annesley & Clayton, 2009). Deferm et al. used mannitol clearance to measure GFR in asphyxiated neonates under therapeutic whole‐body hypothermia. In that study, mannitol concentrations in plasma were measured with mass spectrometry at the same laboratory and with the same method as used in the present study (Deferm et al., 2021). A detailed description of the method used at the laboratory to measure plasma mannitol concentration is found in Supplemental File 2.

The results of the present study and the previous study from our research group (Kiss et al., 2018), suggest that mannitol clearance could be useful for assessment of GFR in patients with CKD. Mannitol is a widely available, inexpensive, and safe medication with minimal adverse effects, making it a practical alternative for GFR measurements. It could also be used as an alternative to iohexol clearance in situations where it might not be appropriate to use iohexol, for example, in known severe allergy to contrast agents or in patients who have been exposed to contrast agents within 5 days of need for clearance measurement.

Our study showed that there was a good agreement between the reference method (multi‐sample iohexol clearance) and the one‐sample mannitol clearance with samples taken 5 and 22 h after the bolus injection of mannitol. The biases were low, and the errors were in the range of 20–24%. Furthermore, the accuracies for P30 and P10 were well above the recommended, with P30 levels of 95‐100% and P10 levels of 60–80% for samples taken at 5 and 22 h, respectively. However, the slightly lower P10 accuracy indicates that further optimization is required before mannitol clearance can be considered a fully equivalent alternative to iohexol clearance. The small sample size of the present study may have increased the sensitivity of accuracy estimates to individual outliers. Therefore, larger studies are warranted to confirm these findings and to refine the one‐sample approach for clinical use.

Currently, there is no standardized dosage protocol for mannitol clearance measurement. To ensure good reproducibility of measured concentrations, we used the lowest therapeutic dose of 0.25 g/kg via intravenous infusion. As mass spectrometry is a highly sensitive method for analyzing plasma concentrations of small molecules, future studies should explore the lowest optimal dose of infused mannitol that maintains the reliability of repeated measurements.

A limitation of the present study was that the patient cohort of the study was small, which may limit the generalizability of the findings. Furthermore, clearance of mannitol was only compared with one reference method, iohexol clearance, as iohexol is the only available standard clearance marker in our country. In our previous study (Kiss et al., 2018), the multi‐sample mannitol clearance was compared with one‐sample ^51^Cr‐EDTA clearance in patients with normal to moderately reduced kidney function with a mean clearance of 60 mL/min/1.73 m^2^. The strength of this study is that the multi‐sample mannitol clearance was compared to a simultaneously performed multi‐sample iohexol clearance in patients with advanced CKD.

In conclusion, this study demonstrated a close agreement between mannitol and iohexol clearance in patients with CKD stages 3–4, particularly when using the multi‐sample approach. The one‐sample mannitol clearance method also showed promising results, though slightly lower accuracy suggests that further refinement and validation of the method is needed. While these findings indicate that mannitol has a potential as an alternative clearance marker for GFR measurement, the evidence remains preliminary. Larger studies across a broader range of kidney function are required to confirm these results and to establish standardized protocols for mannitol dosing, sampling, and analysis before its wider clinical application can be recommended.

## CONFLICT OF INTEREST STATEMENT

The authors declare no conflicts of interest.

## Supporting information

Supplemental file 1.

Supplemental file 2.

## Data Availability

The data that support the findings of this study are available from the corresponding author upon reasonable request.
