# Supplementary material for: Clearance of mannitol for assessment of glomerular filtration rate in chronic kidney disease: A validation against iohexol clearance
Source: Clin Physiol Funct Imaging. 2026 Apr 3;46(3):e70059. doi: 10.1111/cpf.70059 (PMC13047722; doi:10.1111/cpf.70059)
Supplement: Supplementary file 1 — Supplemental file 1. [file CPF-46-0-s001.docx]

**Supplemental file 1**

Extraction of D-mannitol from human heparin plasma samples was performed using protein precipitation followed by derivatization. The obtained derivatives were analyzed using liquid chromatography-mass spectrometry (API 4000 LC-MS/MS). The analyses were performed in accordance with the method described in AWI 4228^1^. The method has been qualified in Ardena Bioanalytical Laboratory study 16366^2^ regarding response function, selectivity, carryover, precision, accuracy, matrix effect, bench-top stability at processing temperature, re-injection stability in the autosampler at 10°C, F/T stability, extracted plasma sample stability, mutual interference of analytes and internal standards, potential interference of coadministered drugs and batch size determination.

References

1. AWI 4228 (current version), entitled: “D-mannitol in human heparin plasma using an (API 4000 LC-MS/MS) system”.
2. M.J. Dröge: “Determination of D-mannitol in human heparin plasma samples using LC-MS/MS”, Ardena Bioanalytical Laboratory qualification study 16366.
