# Supplementary material for: Clearance of mannitol for assessment of glomerular filtration rate in chronic kidney disease: A validation against iohexol clearance
Source: Clin Physiol Funct Imaging. 2026 Apr 3;46(3):e70059. doi: 10.1111/cpf.70059 (PMC13047722; doi:10.1111/cpf.70059)
Supplement: Supplementary file 2 — Supplemental file 2. [file CPF-46-0-s002.docx]

**Supplemental file 2**

**Calculation of Absolute and Relative Iohexol and Mannitol Clearance**

Calculation of Absolute Iohexol/Mannitol Clearance (GFR_abs) from Multiple-Sample Blood Sampling - according to Brøchner-Mortensen, 1972.

Data from sampling time after injection (minutes) and iohexol/mannitol concentration (mg/L) are entered into a diagram where

y = ln(iohexol/mannitol concentration) and x = time from injection

A linear regression line is fitted to the points, and the slope (k) and intercept (m) are calculated according to the formula

y = kx + m

From this, the area under the curve (AUC) is determined:

AUC = (-e^m) / (1000 × k)

Then, an uncorrected absolute GFR (GFR1) is calculated where the dose is the amount of injected iohexol/mannitol (mg):

GFR1 = dose / AUC

GFR_abs is then calculated by correcting GFR1 using the following formula:

GFR_abs = 0.991 × GFR1 - 0.00122 × GFR1^2

Calculation of Relative (Body-Normalized) Iohexol/mannitol Clearance (GFR_rel)

Relative GFR (GFR_rel) is calculated by adjusting GFR_abs against a body surface area (BSA) of 1.73 m²:

GFR_rel = (GFR_abs × 1.73) / BSA

BSA (m²) is calculated according to Haycock et al. (1978) from the patient's height (cm) and weight (kg).

BSA= height(cm)^0.3964 × weight(kg)^0.5378 × 0.024265
